# Dynamic involvement of the core gut microbiome XNP_Guild1 in the evolution of gestational diabetes mellitus

**DOI:** 10.1080/19490976.2026.2623353

**Published:** 2026-01-31

**Authors:** Hualongyue Du, Qiaoxi Lin, Xiaojing He, Bin Yang, Yiyao Huang, Qianbei Li, Yudi Wang, Ruijing Wen, Wenlong Lin, Shenghui Li, Lei Zheng, Zihao Ou

**Affiliations:** a Department of Laboratory Medicine, Nanfang Hospital, Southern Medical University, Guangzhou, People's Republic of China; b Guangdong Provincial Clinical Research Center for Laboratory Medicine, Guangzhou, People's Republic of China; c Center for Infectious Diseases, Vision Medicals Co., Ltd., Guangzhou, People's Republic of China; d Puensum Genetech Institute, Wuhan, People's Republic of China

**Keywords:** Gestational diabetes mellitus, gut microbiome, core microbial guild, grey zone, biomarker

## Abstract

Integrated large-scale maternal microbiome cohort analyses are critical for understanding the development of gestational diabetes mellitus (GDM) and its impact on maternal and offspring health. Here, we analyzed the microbiomes of 2,717 mothers and infants from 9 global cohorts, including both public datasets and a prospective cohort in China, using high-throughput sequencing and multilayer network modeling. We systematically identified and characterized a group of “predicted grey zone” individuals whose gut microbial network features fell between those of healthy and GDM subjects, which represent dynamic ecological transition states in disease development. Notably, we identified and validated across cohorts a core gut microbial guild (XNP_Guild1) that remained highly stable and functionally cohesive across healthy, grey zone, and GDM states, and was significantly associated with both disease progression and early pregnancy risk. In an exploratory intergenerational network analysis, we estimated the vertical transmission effect of the core guild and its potential influence on neonatal growth outcomes. These findings highlight the tight interconnection among core functional gut microbes, transitional ecological states, disease evolution, and maternal–infant health, providing a foundation for future targeted interventions and mechanistic studies of the maternal–offspring microecosystem in GDM.

## Introduction

Gestational diabetes mellitus (GDM) is a prevalent metabolic disorder that poses a serious threat to the health of pregnant women and their offspring worldwide.^1^ The incidence of GDM has increased by more than 30% over the past two decades, becoming the leading cause of pregnancy complications in several countries.^2^ GDM increases the risk of adverse outcomes such as gestational hypertension, macrosomia, preterm birth, and cesarean section,^3^ and is strongly associated with long-term risks of type 2 diabetes and cardiovascular disease in mothers,^4^ as well as irreversible consequences for offspring, including obesity, diabetes, and neurodevelopmental disorders.^5^ Despite standard management during pregnancy, long-term metabolic and developmental abnormalities in GDM mother–infant pairs often persist, underscoring the urgent need for earlier and more precise risk prediction and intervention.^6^ However, early screening and individualized stratification remain limited by the lack of effective tools.^7^ Current clinical prediction largely relies on oral glucose tolerance tests (OGTT) and traditional metabolic indices in the second or third trimester, which rarely identify high-risk individuals before clinical manifestation, resulting in missed opportunities for prevention.^8^ This limitation stems from the complex, dynamic, and heterogeneous pathogenesis of GDM.

Beyond genetic and lifestyle factors, host–microbiome interactions have emerged as a novel framework for understanding GDM risk and progression.^9^ Recent animal and human studies have established the pivotal role of microbiome in regulating metabolic homeostasis, inflammation, and immunity during pregnancy.^10^ Importantly, gut microbes show clear compositional and functional alterations in GDM, and these altered microbial communities can further disturb glucose metabolism and promote disease progression.^11–14^ Nevertheless, these findings are largely derived from cross-sectional, single-time-point, or geographically restricted cohorts, and microbial biomarkers show poor reproducibility both across and within populations over gestation.^15^ Most studies have focused on differences in microbiota composition, failing to address dynamic disease transitions, network remodeling, and underlying ecological drivers.

Crucially, GDM is not a binary or static disease but involves latent, early-stage risk and a continuum of maternal–infant interactions.^16^ Many studies overlook the transitional states between health and disease, limiting the identification of subtypes and dynamic risk stratification.^17^ In addition, most research on maternal and neonatal microbiomes has emphasized compositional similarity or diversity loss, without resolving the functional roles, network properties, or intergenerational transmission of core microbial guilds.^18^ The lack of systematic characterization of dynamically stable and generalizable core microbiota, and their network-level evolution, remains a major obstacle to early screening, precision intervention, and mechanistic breakthroughs in GDM.^19^

In this study, we integrated high-throughput sequencing, machine learning, and ecological network analysis across a global cohort of 2,717 mother and infant microbiome, including 2,655 public samples from eight international cohorts and 62 participants recruited from Guangzhou and Zhangzhou, China. By analyzing gut microbiome profiles spanning healthy, GDM, and intermediate ‘grey zone’ states—defined *via* probabilistic machine learning—we mapped dynamic microbiome changes throughout disease progression. We identified and validated a highly stable, functionally cohesive core microbial guild (XNP_Guild1), conserved across all states. Iterative clustering revealed its ecological and functional coherence. XNP_Guild1 precisely predicted GDM risk, captured key network remodeling in transitional states, and exerted intergenerational functional effects on neonates, impacting early-life gut ecology and growth. These findings establish dynamic core microbiota as central to GDM progression and network stability, and highlight their potential as early biomarkers and intervention targets for maternal and neonatal health.

## Results

### Digestive tract microbial diversity and structural features in GDM

**Figure 1.**
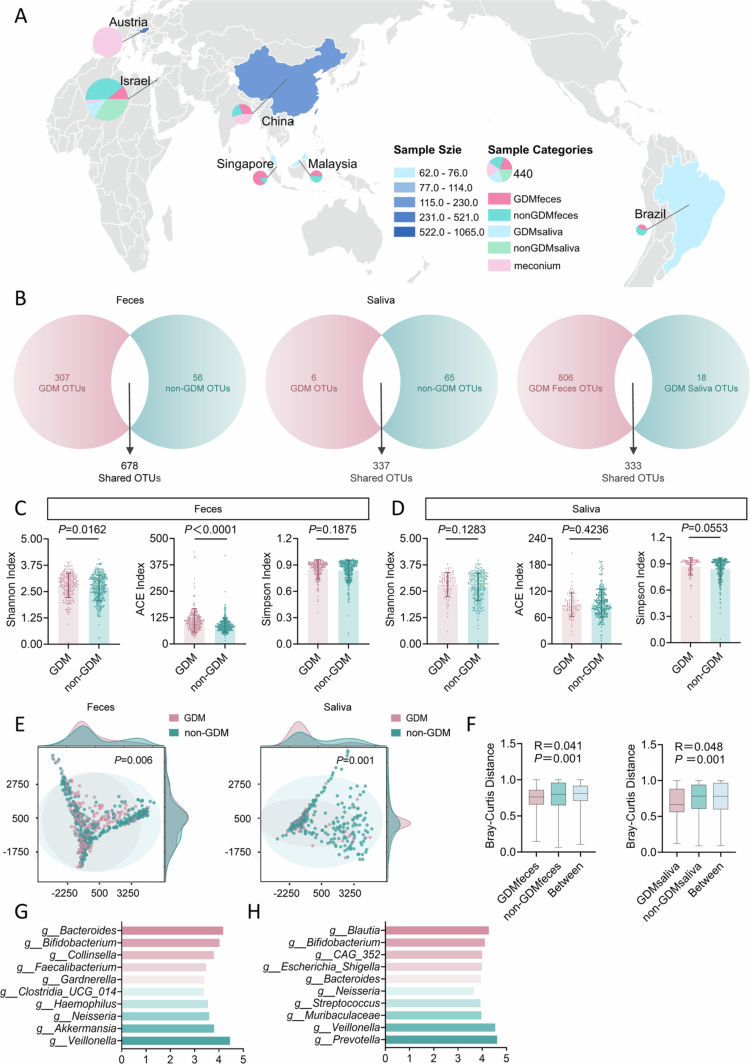
Diversity and composition of digestive tract microbiota in GDM. A, Geographic distribution and types of pregnancy-related microbiome samples included in this study. B, Venn diagram illustrating substantially greater diversity of operational taxonomic units (OTUs) in fecal compared to salivary samples, and the distribution of GDM-specific and control-specific OTUs. C, Alpha diversity (Shannon and ACE indices) of gut microbiota in fecal samples from GDM (*n* = 222) and non-GDM (*n* = 366) groups. D, Alpha diversity of gut microbiota in salivary samples from GDM (*n* = 88) and non-GDM (*n* = 256) groups. E, Principal coordinates analysis (PCoA) depicting beta diversity differences between GDM and non-GDM groups in fecal (left) and salivary (right) samples. F, Analysis of similarities (ANOSIM) showing significant community structure differences between GDM and non-GDM in both sample types. G, H, Key differentially abundant bacterial taxa in fecal (G) and salivary (H) samples identified by linear discriminant analysis effect size (LEfSe). Data are presented as mean ± s.d. in box plots; boxes indicate interquartile range, horizontal lines denote the median, and whiskers extend to the minimum and maximum values (C, D, F). Statistical analyses were performed using two-tailed unpaired t-tests (C, D), permutational multivariate analysis of variance (E), or ANOSIM (F). R values for ANOSIM were determined by non-parametric permutation tests. GDM, gestational diabetes mellitus.

To investigate the potential association between the human microbiome and GDM, we systematically analyzed 16S rRNA sequencing data from the digestive tract—the most microbiota-rich organ system—using saliva as a proxy for the upper gastrointestinal tract and feces for the lower tract. This study integrated data from 8 public datasets worldwide, including a total of 2,655 samples from pregnant women and infants (**Extended Data Tables 1–8**). After stringent quality control and exclusion of samples with sequencing depth below 10,000 reads, 908 maternal fecal, 441 maternal salivary, and 378 infant fecal samples were retained for downstream analysis (Figure 1A).

A total of 1995 operational taxonomic units (OTUs) were identified across maternal fecal and salivary samples. Among these, 678 OTUs were detected in fecal samples, with GDM-specific OTUs numbering 307−5.48 times more than in non-GDM controls (Figure 1B), indicating markedly increased colonization of novel gut microbes in GDM. In salivary samples, 337 OTUs were identified, with only 6 being GDM-specific, compared to 65 in non-GDM individuals (Figure 1B), suggesting that GDM mainly affects the oral microbiome by reducing existing taxa or their abundance. There were 333 OTUs shared between fecal and salivary samples in GDM patients, but the number of fecal-specific OTUs reached 806, about 45 times that of saliva (Figure 1B), further highlighting the higher microbial richness in the gut compared to the oral cavity during pregnancy.

Alpha diversity analysis revealed that the Shannon and ACE indices were significantly higher in fecal samples from GDM than from controls (Figure 1C), whereas no significant differences were observed between groups in salivary samples (Figure 1D). Principal coordinates analysis (PCoA) showed distinct clustering of microbial community structures between GDM and non-GDM groups in both fecal and salivary samples (Figure 1E). Consistently, ANOSIM analysis confirmed statistically significant differences in community structure between groups in both fecal (R = 0.041, *P* = 0.001) and salivary samples (R = 0.048, *P* = 0.001; Figure 1F), indicating that GDM alters both gut and oral microbiota, with a stronger association in the gut.

To further explore dominant taxa, we visualized bacterial taxonomic tree in GDM and control groups. In saliva, GDM primarily led to decreased abundance of commensal taxa, with a limited increase in certain pathogenic groups, reflecting a loss-driven alteration of the oral microbiome (**Extended Data Figure 1**). In contrast, the fecal microbiota of GDM displayed more complex ecological remodeling, involving substantial alterations across multiple phyla and subordinate taxa (**Extended Data Figure 2**). Several metabolism- and inflammation-associated taxa were significantly enriched in GDM fecal samples, suggesting a central role of the gut microbiome in GDM pathogenesis (**Extended Data Figure 2**). LEfSe analysis revealed distinct metabolic and inflammation-associated taxa enriched in both fecal and salivary samples of GDM. *Bifidobacterium* and *Bacteroides* were consistently elevated in GDM samples, while Collinsella—linked to metabolic dysregulation—was unique to GDM fecal microbiota (Figure 1G). In the salivary microbiome, *Blautia* and *Escherichia-Shigella*, taxa associated with inflammation, were enriched in GDM (Figure 1G). Collectively, these results suggest that gut microbial alterations may play a more prominent role in the pathophysiology of GDM.

**Figure 2.**
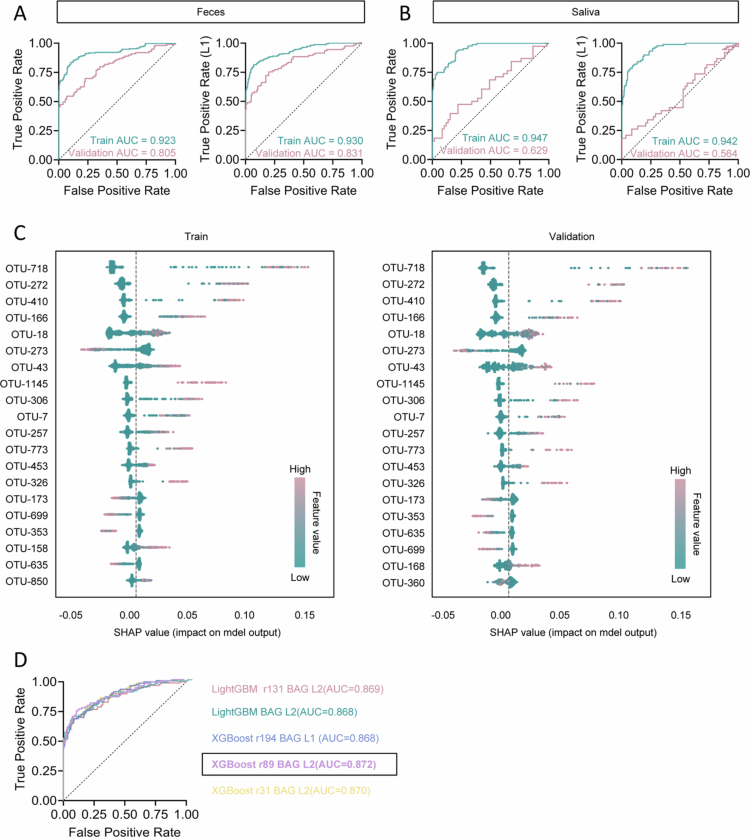
Machine learning-based identification of gut and salivary microbial signatures associated with GDM. A, Receiver operating characteristic (ROC) curves for Random Forest (RF, left) and L1-regularized RF (L1+RF, right) models applied to fecal microbiota training and validation cohorts. B, ROC curves for RF (left) and L1+RF (right) models applied to salivary microbiota training and validation cohorts. C, SHapley Additive exPlanations (SHAP) summary plots of the top 20 features in the L1+RF model for GDM fecal training (left) and validation (right) cohorts. SHAP values reflect each feature’s impact on model output; positive values indicate increased predicted GDM risk. Each dot represents a sample, colored by the corresponding feature value, with vertical stacking indicating density. D, ROC curve depicting the performance of the best AutoGluon-selected ensemble model (XGBoost r89 BAG L2) for the fecal microbiota training and validation cohorts.

### Machine learning-based identification of key gut microbial features associated with GDM

To further investigate the association between the gut microbiome and GDM, we randomly divided the fecal and salivary microbiome datasets into training and validation cohorts at a 7:3 ratio, and systematically evaluated the performance of multiple machine learning algorithms for GDM classification. A standard random forest (RF) model achieved robust performance on the independent validation set, with an area under the receiver operating characteristic curve (AUC) of 0.805 (Figure 2A). Incorporation of L1 regularization (L1 = 0.1) to select informative features further improved the validation AUC to 0.831 in the L1+RF model by reducing noise and enhancing generalizability (Figure 2A). In contrast, models based on salivary microbiome data yielded a lower validation AUC of 0.629 and 0.564 (L1+RF model) (Figure 2B), underscoring the predominant role of the gut microbiome in GDM pathophysiology.

To delineate the contribution of specific bacterial features, we applied SHapley Additive exPlanations (SHAP) analysis to the L1+RF model. The top 20 OTUs contributing to model performance were highly consistent across training and validation sets (Figure 2C), with OTU-718, OTU-272, OTU-410, OTU-166, and OTU-18 among the most influential variables. These OTUs demonstrated stable and significant predictive contributions across datasets. Notably, several OTUs (e.g., OTU-718 and OTU-272) exhibited stronger associations with GDM prediction when present at higher abundance (Figure 2C), suggesting their potential role in GDM-related microbial dysbiosis.

To further confirm the robustness of the selected gut microbial features associated with GDM, we utilized the AutoGluon automated machine learning framework to systematically evaluate the performance of multiple ensemble algorithms and different bagging layers (L1, L2). Receiver operating characteristic analysis showed that all models achieved high AUC values in the validation set, reflecting consistent and robust classification performance (Figure 2D). Importantly, we observed that the key microbial features identified for GDM discrimination were highly concordant across diverse statistical models and ensemble approaches, including XGBoost, LightGBM, and random forest, regardless of parameter settings or bagging layers (Figure 2D). This high degree of feature overlap across models provides strong evidence for the stability and robustness of the selected gut microbial signatures, independent of the specific modeling strategy employed.

### Prediction “grey zone” and unique ecological network features in GDM

**Figure 3.**
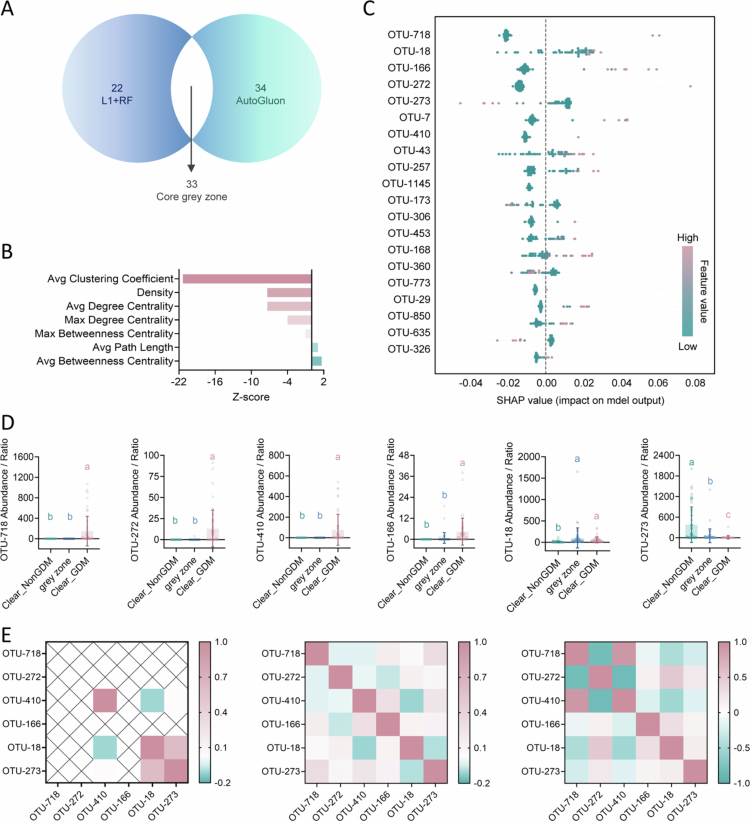
Identification and characterization of a “prediction grey zone” with distinct ecological features in GDM. A, Venn diagram showing the overlap of samples in the prediction grey zone as identified by two high-performing machine learning models (L1+RF and AutoGluon). B, Z-scores of key network metrics in the grey zone compared to random networks. Positive Z-scores (right) indicate higher-than-expected topological properties, while negative values (left) indicate lower-than-expected values, highlighting a distinct ecological structure within the grey zone. C, SHAP summary plot showing the top 20 microbial features contributing to the L1+RF model’s predictions for grey zone samples. D, Abundance dynamics of representative OTUs across Clear_NonGDM, grey zone, and Clear_GDM groups. Distinct letters indicate statistically significant differences between groups (*P* < 0.05; Kruskal–Wallis test, *n* = 53 (Clear_NonGDM), *n* = 55 (grey zone), *n* = 46 (Clear_GDM)). Data are presented as mean ± s.d. E, Heatmaps illustrating the changing abundance and correlation patterns of selected opportunistic pathogens in fecal samples from Clear_NonGDM (left) to grey zone (middle) to Clear_GDM (right).

Although machine learning models demonstrated strong performance in GDM classification, a subset of clinically diagnosed individuals in the independent validation cohort (*n* = 55; 26 GDM, 29 non-GDM) exhibited predicted GDM probabilities between 0.4 and 0.6, where the L1+RF model showed the highest misclassification rates of 51% in the 0.4–0.5 probability bin and 83.3% in the 0.5–0.6 bin (**Extended Data Table 9**). This group constituted a “prediction grey zone” with ambiguous boundaries. To determine whether this phenomenon was attributable to model-specific biases, we re-evaluated the data using the AutoGluon platform. AutoGluon not only reproduced the grey zone but also identified a set of samples highly overlapping with those defined by the L1+RF model (33 cases; Figure 3A). This cross-model concordance indicates that the grey zone is unlikely to be random noise, but instead may reflect genuine biological heterogeneity, providing crucial insight into early or transitional states of GDM.

To investigate whether the grey zone possesses distinct ecological network features, we defined samples with predicted probabilities >0.7 and clinical GDM diagnosis as Clear_GDM, and those with probabilities <0.3 and non-GDM diagnosis as Clear_NonGDM. Using a combined background set including all grey zone, Clear_GDM, and Clear_NonGDM samples, we conducted 1,000 random node subset permutations and permutation tests to compare the structural properties of the grey zone network with random networks. The grey zone network exhibited significantly lower mean clustering coefficient (Z = –21.36), network density (Z = –7.37), and maximal degree centrality (Z = –4.01) (Figure 3B), indicating a uniquely sparse, low-modularity structure lacking core nodes—characteristics of an unstable ecological transition state between health and disease.

We further analyzed key bacterial features within the grey zone and compared the dynamic changes of representative OTUs among Clear_NonGDM, grey zone, and Clear_GDM groups (Figure 3C). In the healthy group, OTU-273 (*Akkermansia*) and OTU-18 (*Lachnoclostridium*) displayed strong positive correlation, a relationship that disappeared in both the grey zone and GDM groups (Figure 3D). Instead, new pathological modules emerged, dominated by opportunistic pathogens such as OTU-718 (*Finegoldia*) and OTU-410 (*Anaerococcus*) (Figure 3E). Together, these results suggest that the prediction grey zone is not a model artifact but may represent a dynamic, highly heterogeneous transitional stage in the ecological progression from health to GDM. Thus, GDM development appears to follow a continuum of ecological transitions rather than a simple binary switch.

### Identification and functional characteristics of the stable core guild XNP_Guild1 during GDM progression

Most current studies focus on binary differences between health and disease, yet whether a stable core gut guild persists during the ecological transition from health to GDM remains unknown.^20^ To address this, we constructed co-occurrence networks of gut microbial OTUs for both GDM and non-GDM groups. GDM network (211 nodes, 5,199 significant edges, density 0.2347) displayed substantially reduced connectivity and density (**Extended Data Figure 3**) compared to the non-GDM network (197 nodes, 6,054 significant edges, density 0.3136) (**Extended Data Figure 4**), along with a marked decrease in the proportion of negative edges (**Extended Data Table 10)**. These changes reflect profound restructuring of microbial associations and ecological niches during GDM progression.

**Figure 4.**
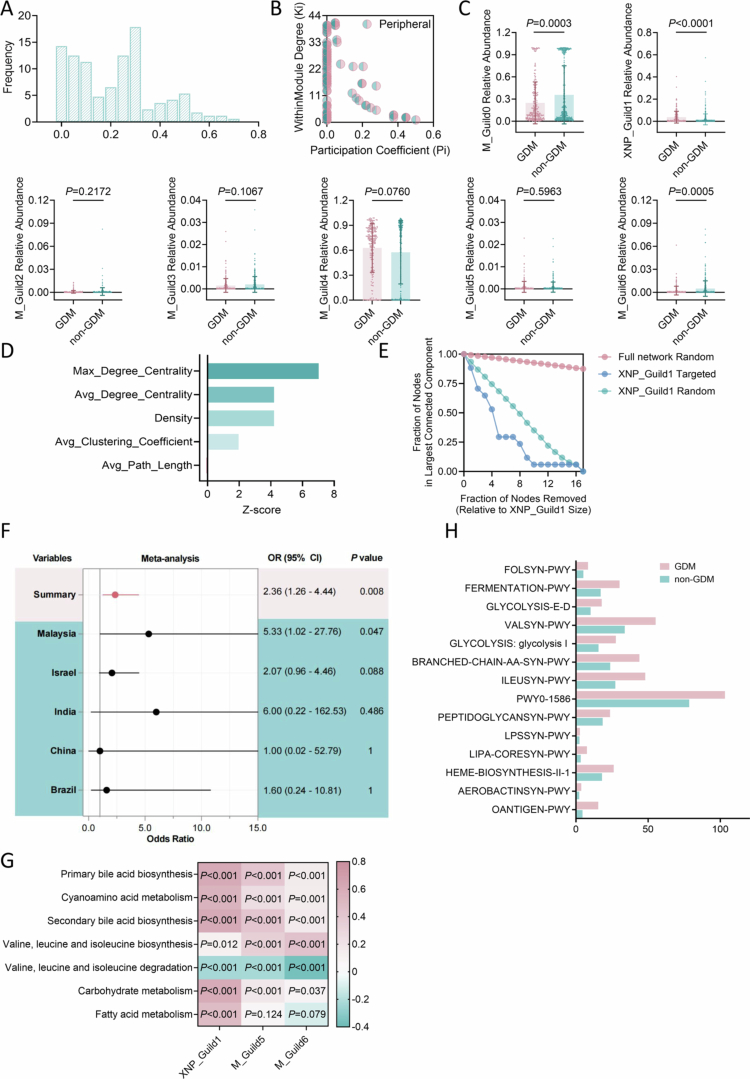
Identification and functional characterization of a stable core gut microbial guild (XNP_Guild1) associated with GDM. A, Histogram showing the distribution of microbial community connectivity (“sociality”) based on the Jaccard index in GDM and non-GDM fecal samples. B, Zi-Pi plot illustrating the modular organization of the stable gut microbial network in GDM, with most nodes displaying low participation coefficients, indicative of strong within-guild cohesion. C, Relative abundance of the seven core microbial guilds identified in the stable network, comparing GDM (*n* = 222) and non-GDM (*n* = 366) groups. XNP_Guild1 is significantly enriched in GDM. *P* values are from two-tailed statistical tests. D, Z-scores of topological network metrics for XNP_Guild1, showing that this guild exhibits a highly stable, non-random structure compared to randomized networks. E, Robustness analysis of XNP_Guild1, showing that targeted removal of guild members dramatically reduces the integrity of the largest connected component, indicating strong network resilience dependent on XNP_Guild1. F, Forest plot showing the association between XNP_Guild1 abundance and GDM risk across independent external cohorts from five countries. Odds Ratio and 95% confidence intervals (CIs) were estimated by univariate analysis. G, Heatmap depicting correlations between XNP_Guild1 abundance and key metabolic pathways relevant to GDM, with a strong positive correlation observed with BCAA biosynthesis. H, Differential abundance of major functional pathways of *E. coli* between GDM and control groups, based on metagenomic functional profiling.

We next identified a set of OTUs consistently present and correlated in both networks, forming a “stable network”, and further applied community detection algorithms to delineate seven core functional guilds (**Extended Data Figure 5**). Topological analysis showed this network was highly modular, with all node participation coefficients (Pi) below 0.62 (Figure 4A,B), indicating that nearly all nodes were tightly confined within their own guild—demonstrating both statistical and structural cohesion. Among these, a core guild composed of 17 OTUs was highly conserved across both health and GDM states and was significantly enriched in GDM (Figure 4C). Given its stability and functional synergy during disease evolution, we designated this group XNP_Guild1 (**Extended Data Table 18**).

**Figure 5.**
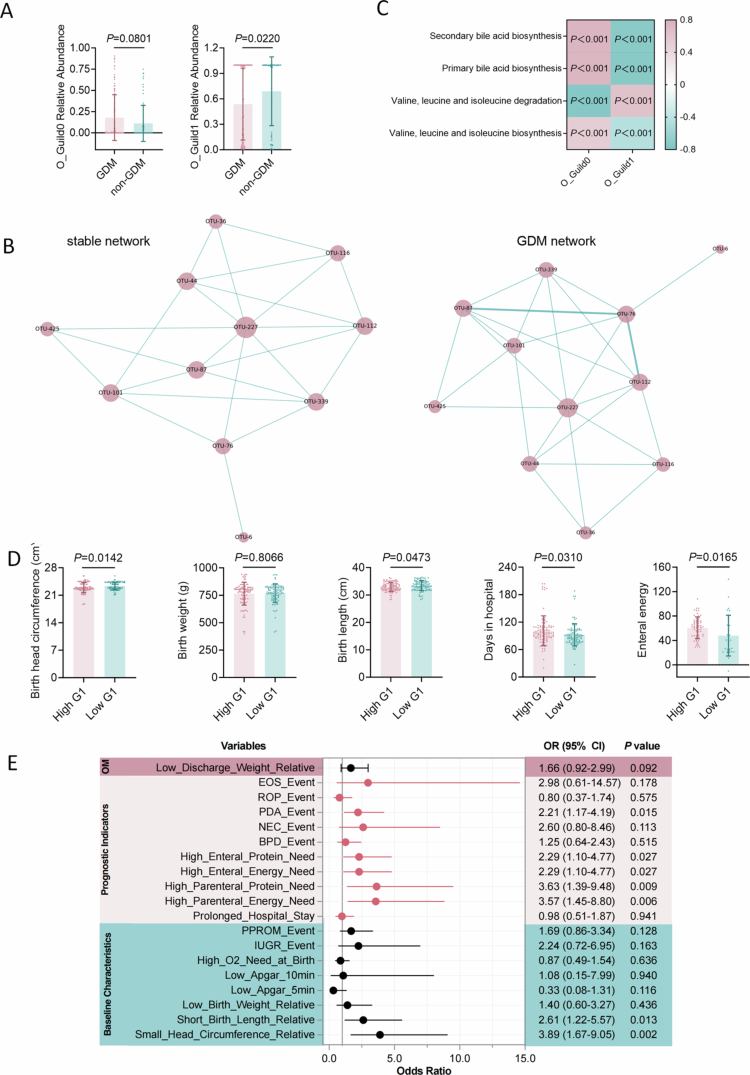
Vertical transmission and early-life impact of GDM-associated core gut guild XNP_Guild1 in offspring. A, Relative abundance of meconium O_Guild0 and O_Guild1 in neonates born to GDM and non-GDM mothers, with Guild 0 significantly enriched in GDM (*n* = 99 (GDM), *n* = 69 (non-GDM)). B, Network structures of O_Guild0 in GDM and non-GDM offspring, demonstrating preserved internal connectivity and stability in the GDM. C, Heatmap illustrating correlations between O_Guild0 abundance and GDM-related metabolic pathways in neonates, closely paralleling the functional characteristics of maternal XNP_Guild1. *P* values derived from correlation analysis, all *P* < 0.001. D, Impact of high versus low neonatal XNP_Guild1 abundance on early prognostic indicators among extremely preterm infants (*n* = 105 (High G1), *n* = 105 (Low G1)), including head circumference, birth length, days in hospital, and enteral energy requirements. *P* values from two-tailed unpaired t-tests. E, Forest plot showing the association between neonatal XNP_Guild1 abundance and clinical outcomes, including early growth, need for intensive care, and risk of adverse events. Odds ratios (ORs) and 95% confidence intervals (CIs) estimated by univariate analysis. Significant associations observed for reduced head circumference, short birth length, and increased parenteral nutrition requirements in the high-abundance group. Statistical analyses were performed using two-tailed unpaired t-tests (A, D, E) or multivariable linear regression and univariate analysis as indicated (F). Data are presented as mean ± s.d.

Detailed analysis revealed strong internal co-occurrence among XNP_Guild1 members, with increased connectivity and network density in GDM, including 11 new exclusive edges and enhanced synergistic effects (**Extended Data Figure 6A**). OTU-46 (*Escherichia*) emerged as the key hub, displaying the highest degree and betweenness centrality (**Extended Data Figure 6B and Extended Data Table 11**). In GDM, XNP_Guild1 underwent extensive rewiring, with diminished links to potential probiotics and strengthened associations with opportunistic pathogens (**Extended Data Figures 7 A and 7B**), resulting in a more tightly knit ecological module. Topological metrics confirmed that XNP_Guild1 exhibited significantly higher network density, mean degree centrality, maximal degree centrality, and clustering coefficient than randomized networks, highlighting its highly ordered, non-random structure (Figure 4D). Robustness tests further indicated that targeted removal of the most central nodes (with the highest degree centrality) within XNP_Guild1, particularly *Escherichia*, markedly impaired the overall resistance of XNP_Guild1 to perturbation, underscoring the guild’s high dependence on the integrity of its core members. (Figure 4E).

**Figure 6.**
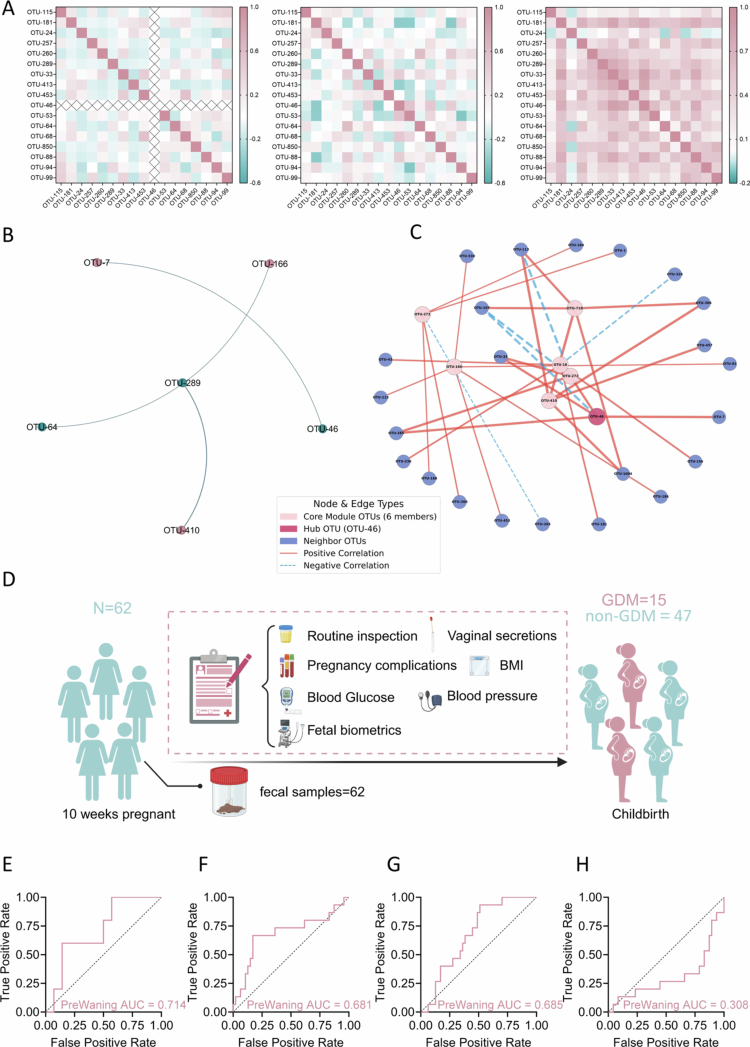
XNP_Guild1 drives the microbial network transition and enables early prediction of GDM. A, Heatmap of intra-guild correlations among XNP_Guild1 members across different states, highlighting the grey zone as a transitional or high-risk state for GDM. B, Network diagrams showing changes in the interaction network between XNP_Guild1 and key grey zone OTUs during the transition from health to GDM. C, Social interaction network of grey zone indicator OTUs and core member OTU-46 (*Escherichia*), illustrating the emergence of pathogenic modules. D, Flowchart of sample and data collection for the early pregnancy warning cohort. The flowchart was created with BioRender (https://www.biorender.com). E–H, ROC curves for GDM prediction in the early warning cohort, comparing models based on: E, Logistic regression using XNP_Guild1 features; F, Best-performing AutoGluon ensemble model (XGBoost_r95_BAG_L2_FULL); G, L1-regularized random forest (L1+RF) model; H, L1+RF model after removal of XNP_Guild1 features. Abundance data are shown as mean ± s.d. Statistical analysis was performed using two-tailed unpaired t-tests (E).

To validate the stability of XNP_Guild1, we confirmed its consistent enrichment in GDM using independent external cohorts (**Extended Data Table 12 and Extended Data Figure 8A**)** **and meta-analysis across five countries (I² = 0.0%), with high-abundance individuals having 2.36-fold higher odds of GDM (Figure 4F). Elevated XNP_Guild1 abundance in GDM was observed across Israeli, Brazilian, and Malaysian populations, reaching significance in Israel and Malaysia (**Extended Data Figure 8B**). Longitudinal analysis revealed that XNP_Guild1 was stably present in early pregnancy, peaked in late pregnancy, and declined postpartum, tracking closely with GDM risk progression (**Extended Data Figure 8C**).** **Collectively, XNP_Guild1 represents a stable, highly conserved, and functionally synergistic core gut guild throughout GDM evolution, providing a new mechanistic and predictive target for early detection and intervention.

Functional prediction further showed that XNP_Guild1 abundance correlated positively with fatty acid metabolism, branched-chain amino acid synthesis, and bile acid metabolism, but negatively with BCAA degradation pathways (Figure 4G), suggesting it may promote GDM progression by orchestrating multiple metabolic routes. To explore its mechanistic role, we leveraged a Chinese Metagenomics-based cohort (**Extended Data Table 13**) and found that *Escherichia* (OTU-46), as the key hub, was associated with elevated pathways linked to outer membrane biosynthesis, virulence, core metabolism, and survival in GDM (Figure 4H). These findings indicate that GDM gut environment may favor the growth and metabolic activity of these pathobionts, exacerbating disease via endotoxin release, inflammation, and metabolic disruption. We also examined OTU-273 (*Akkermansia*) and OTU-18 (*Lachnoclostridium*), which formed tight associations in the clear_GDM group. HUMAnN3 pathway analysis showed *Akkermansia* actively proliferated in GDM, implicating substantial changes in mucosal barrier dynamics (**Extended Data Figure 8D**), while Lachnoclostridium exhibited reduced activity, with suppressed metabolism and SCFA production (**Extended Data Figure 8E**). Together, these data support the existence of a stable, functionally relevant core guild—XNP_Guild1—intimately linked to the onset and progression of GDM.

### Intergenerational transmission of the core guild XNP_Guild1 and its impact on early offspring health outcomes

Given the potential for GDM to exert irreversible effects on offspring,^21^ we further investigated whether the core gut guild XNP_Guild1 could be vertically transmitted and influence the early gut microbiota and clinical outcomes in neonates. Using fecal samples from 168 GDM and non-GDM offspring, we constructed co-occurrence networks for each group (**Extended Data Table 14**). The gut microbial network of GDM offspring exhibited markedly reduced density (0.3) (**Extended Data Figure 9**) compared to that of the non-GDM group (0.5) (**Extended Data Figure 10**), indicating a sparser microbial interaction network in early life, consistent with observations in maternal GDM samples.

Applying a co-occurrence consistency approach, we identified a stable network across both groups (**Extended Data Figure 11**), revealing two distinct functional guilds. O_Guild0 was enriched in GDM offspring, while O_Guild1 predominated in non-GDM offspring (Figure 5A). Notably, O_Guild0 retained a stable core structure in GDM (Figure 5B). Of particular interest, OTU-46 (*Escherichia*)—the central hub of XNP_Guild1 in maternal GDM—was also classified as a key member of GDM Network in neonatal fecal networks, exhibiting high degree and betweenness centrality (betweenness = 0.384615, degree centrality = 0.034) in GDM offspring (**Extended Data Table 15**). Furthermore, in both maternal GDM and GDM offspring samples, O_Guild1 and O_Guild0 showed a strong negative correlation with BCAA degradation pathways and a strong positive correlation with bile acid biosynthesis (Figure 5C), mirroring the functional profile of XNP_Guild1 in maternal GDM. These findings suggest that XNP_Guild1 represents a core functional module with the capacity for intergenerational transfer, maintaining key metabolic characteristics from mother to child.

To further evaluate the impact of high XNP_Guild1 abundance (High G1) on neonatal outcomes, we analyzed an independent external validation cohort (**Extended Data Table 16**). Although there were no significant differences in Apg5 (**Extended Data Figure 12 A**) and Apg10 (**Extended Data Figure 12B**) between groups, multivariable linear regression showed that neonates with high XNP_Guild1 abundance had significantly lower birth length (mean difference: –0.50 cm, 95% CI: [–0.9955, –0.0131], *P* = 0.044) and head circumference (mean difference: –0.40 cm, 95% CI: [–0.7246, –0.0767], *P* = 0.016) compared to those with low abundance, as well as prolonged hospital stays (mean increase: 9.3 days, 95% CI: [1.08, 17.48], *P* = 0.027; Figure 5C), and required more intensive parenteral nutrition (Figure 5D). Further risk analysis indicated that among extremely preterm infants, high XNP_Guild1 abundance was associated with an increased risk for head circumference below the median (3.89-fold), shorter length (2.61-fold), need for high-energy intravenous nutrition (3.57-fold), and patent ductus arteriosus (2.21-fold) compared to the low-abundance group (Low G1) (Figure 5E). Collectively, these findings demonstrate that XNP_Guild1 is not only a core GDM-associated gut guild with significant intergenerational transmission capacity but is also strongly linked to adverse early growth and developmental outcomes in offspring.

### Early dynamic changes in XNP_Guild1 may drive grey zone evolution and offer early predictive potential for GDM

Given that XNP_Guild1 exhibited alterations as early as the first trimester,^22^ we hypothesized that it may represent a key microbial driver in the onset of GDM. We therefore compared XNP_Guild1 abundance across Clear_NonGDM, grey zone, and Clear_GDM groups. XNP_Guild1 abundance in the grey zone was highly similar to that in Clear_GDM and both were significantly higher than in Clear_NonGDM, indicating that the grey zone likely represents a high-risk or transitional stage in GDM development (**Extended Data Figure 13**).

Next, we analyzed the correlation networks among XNP_Guild1 members. Spearman correlation analysis revealed a marked dynamic shift in network connectivity: member associations were sparse in the healthy state, began to restructure with emergent synergistic patterns in the grey zone, and ultimately formed a dense, highly interactive network in GDM (Figure 6A). This transition indicates that the grey zone already harbors GDM-related microbial features and serves as a critical inflection point in disease evolution. Further topological analysis of the combined network of XNP_Guild1 and critical grey zone variables showed that the network was almost absent in Clear_NonGDM (average degree = 1.0) (**Extended Data Figure 14**), began to assemble in the grey zone (average degree = 1.83) (**Extended Data Figure 15**), and expanded explosively in Clear_GDM (average degree = 14.58, density = 0.634) (**Extended Data Figure 16**). Notably, the mean clustering coefficient increased from 0 to 0.864, while the mean path length decreased to 1.409 (**Extended Data Figure 16**). To specifically quantify the integration of these two groups, we then isolated the sub-network consisting only of the ‘cross-group’ edges (i.e., connections between XNP_Guild1 members and grey zone OTUs). This analysis pinpointed the transition: in the grey zone, this inter-group network was minimal (Average Degree = 1.0) and the two groups remained functionally separate, as indicated by a high modularity (Modularity = 0.646) (Figure 6B). In the Clear_GDM state, however, this bridging network not only expanded (Average Degree = 6.5), but its modularity collapsed to 0.09 (**Extended Data Figure 17**). This dramatic drop in modularity from 0.646 to 0.09 provides quantitative evidence that XNP_Guild1 and the grey zone OTUs had functionally fused, merging from two distinct communities into a single, highly interconnected pathogenic module. These findings suggest that previously isolated OTUs begin to cluster during the grey zone, eventually forming a highly cohesive, functionally active module in GDM. In addition, in the Clear_GDM group, key grey zone OTUs established tight associations with the XNP_Guild1 core member OTU-46 (*Escherichia*), suggesting that interactions between opportunistic pathogens and core guild members may promote GDM onset (Figure 6C).

To further evaluate the early diagnostic potential of XNP_Guild1, we analyzed 200 maternal fecal samples collected in early pregnancy (Figure 6D), with 62 subjects tracked throughout gestation. XNP_Guild1 abundance was elevated in women who developed GDM (**Extended Data Table 17**). A logistic regression model based on XNP_Guild1 member OTU abundance in the early pregnancy cohort achieved an AUC of 0.71 in the validation set, outperforming the best AutoGluon (XGBoost, AUC = 0.681) and L1+RF (AUC = 0.685) models (Figure 6E−G). Notably, removal of XNP_Guild1 features from the L1+RF model led to a precipitous drop in AUC from 0.685 to 0.308 (Figure 6H), effectively abolishing early predictive capability and strongly indicating that XNP_Guild1 is the principal determinant of early GDM diagnostic sensitivity.

Together, these results establish XNP_Guild1 as a stable and functionally active core guild driving the transition from health to GDM *via* the grey zone, orchestrating microbial network remodeling and providing a key biomarker and mechanistic basis for early risk stratification and precision prediction in GDM.

## Discussion

Increasing attention has been directed toward the role of the gut microbiome in the onset, progression, and intergenerational effects of GDM^23^; however, the complexity and high temporal variability of the microbiome have made it challenging to identify key microbial drivers associated with the disease.^24^ Most previous studies have relied on cross-sectional designs and have largely focused on decreases in microbial abundance or diversity,^25^ failing to capture the core ecological dynamics underlying disease evolution. In this study, leveraging large-scale, multi-center maternal and neonatal cohorts and integrating machine learning with ecological network modeling, we systematically characterized the dynamic changes of GDM-associated microbial communities. While the concept of core microbial guilds has previously been proposed in a pan-disease context, our study provides new, disease-specific insights by demonstrating that the assembly and ecological function of these guilds undergo dynamic and GDM-specific remodeling during the development and progression of gestational diabetes mellitus. Unlike prior studies that primarily describe the static presence or absence of core guilds, we reveal that in GDM, the XNP_Guild1 not only remains highly stable across health, transitional, and disease states, but also exhibits intensified internal connectivity and functional synergy as the disease advances. Our integrative network and intergenerational analyses further uncover that this core guild is vertically transmitted from mother to infant and is robustly associated with early-life growth outcomes—a level of mechanistic and clinical linkage not previously reported for GDM. These findings extend the core guild framework from static, cross-sectional associations to a dynamic, temporally resolved, and disease-specific ecological model.

Although increased gut microbial diversity is often considered a hallmark of a healthy microbiome, we and others have observed higher fecal alpha diversity in GDM.^26^ This pattern suggests that diversity expansion under metabolic stress may reflect a dysregulated rather than beneficial ecological state. Our cross-cohort analyses further show that this trend is consistently present across multiple populations, indicating that it is not driven by a single dataset. Existing research on GDM microbiome has largely been limited to static comparisons between healthy and diseased states, with an emphasis on loss of beneficial or dominant taxa.^27^ While some recent studies have employed multi-omics integration and machine learning for prediction, these approaches often remain focused on feature selection, overlooking the complex interaction networks among features and their contributions to disease phenotypes.^28^ To overcome the limitations of traditional diversity and abundance analyses, we employed L1 regularization, random forest, and AutoGluon algorithms to build robust models for GDM discrimination. SHAP value analysis and feature selection not only quantified the individual contribution of OTUs but also identified “prediction grey zone” cases—high-uncertainty samples near the decision threshold. Co-occurrence network and correlation analyses demonstrated that these cases are characterized by fragmented microbial interactions and the absence of key ecological hubs. Unlike prior studies that ignored model uncertainty or excluded ambiguous cases,^29^ our work demonstrates that the grey zone reflects real ecological heterogeneity, providing a conceptual and practical foundation for early, dynamic risk stratification and personalized intervention.

Central to our findings is the identification and characterization of XNP_Guild1—a core functional guild that remains highly stable and synergistic across health, grey zone, and GDM states. Previous literature has highlighted the importance of core microbial taxa in metabolic health, but often at disease endpoints or focusing on individual genera,^30^ without addressing the network-level synergy and temporal evolution.^31^ We demonstrate that XNP_Guild1 is enriched prior to clinical manifestation of GDM, exerts central control over the ecological network, and serves as a major driver of network remodeling and metabolic function. This concept of a “dynamic core guild” moves beyond the traditional paradigm of static differential taxa, offering a new pathway for biomarker discovery, mechanistic research, and targeted intervention. Importantly, the stability and cross-cohort reproducibility of XNP_Guild1 across countries, centers, and longitudinal samples highlight its potential as a broadly applicable indicator, a property rarely addressed in previous studies.

Moreover, we show that the ecological and functional characteristics of XNP_Guild1 can be vertically transmitted from mother to infant, influencing neonatal gut ecology and growth outcomes. Prior research on the maternal–infant microbiome has focused primarily on compositional similarity or loss of diversity^32^; the functional inheritance of core guilds and their clinical implications have not been well explored.^33^ By integrating co-occurrence network and metabolic function analyses, we traced the intergenerational transmission of core functional guilds and linked them to early developmental outcomes in neonates. This not only fills a gap in understanding the relationship between maternal microbiome inheritance and health, but also suggests that optimizing the structure of core microbial networks could be a novel strategy for improving GDM-related maternal and infant outcomes.

We acknowledge several limitations. First, despite the use of multi-center, cross-regional cohorts and multiple analytical approaches to enhance robustness, the observational nature of our study precludes direct inference of causality for core guilds such as XNP_Guild1 in GDM pathogenesis; mechanistic validation using animal models and functional interventions is warranted. Second, technical batch effects across sequencing platforms and processing pipelines may introduce variability; future work should prioritize standardized protocols and cross-platform validation. Third, longer-term follow-up and experimental validation are needed to fully assess the sustained impact of core guilds on maternal and offspring health. Furthermore, while our ‘grey zone’ provides a novel ecological insight, its direct clinical utility is currently limited by the lack of standardized, in-depth clinical metadata in the public datasets analyzed. Future prospective studies correlating this ecological state with deep clinical phenotypes are required to bridge this gap. In addition, the use of 16S rRNA amplicon sequencing limits strain-level resolution, preventing robust inference of vertical transmission and within-species evolution during pregnancy and early life. Finally, the functional profiles in this study were inferred from taxonomic composition and thus reflect predicted potential rather than measured metabolic activity; validation with shotgun metagenomics and other functional assays will be important in future work.

## Conclusion

In summary, using large-scale maternal–infant cohorts and advanced machine learning and ecological network analysis, we provide a comprehensive characterization of dynamic gut microbiome evolution, core guild network structure, and intergenerational transmission in GDM. Our work establishes a new conceptual framework in which a “dynamic core guild” orchestrates microbiome network remodeling and risk propagation, thereby expanding mechanistic understanding and offering actionable paths for early screening, risk stratification, maternal–infant intervention, and broader studies in metabolic disease microbiome research.

## Method

### Sample collection

This study was conducted at Nanfang Hospital, Southern Medical University (Guangzhou, China) and Zhangzhou No. 2 People's Hospital (Zhangzhou, China) between January 2024 and December 2024. A total of 62 pregnant women at 10–12 weeks of gestation were enrolled. Inclusion criteria were: singleton pregnancy, and absence of pre-existing metabolic diseases, chronic gastrointestinal disorders, or major organ dysfunction. Exclusion criteria included: use of antibiotics, probiotics, or bowel-cleansing agents within three months prior to sampling; severe pregnancy complications; or multiple gestation. No specific dietary restrictions were imposed prior to sample collection. Fecal samples were obtained during early pregnancy (10–12 weeks gestation) using sterile, DNase/RNase-free collection tubes, with approximately 200 mg per sample. All samples were transferred to −80 °C within 2 hours of collection. The study protocol was approved by the Ethics Committees of Nanfang Hospital (approval number: NFEC-2024-201) and Zhangzhou No. 2 People's Hospital (approval number: LL2023-21).

### 16S rRNA gene amplification and sequencing

The V3–V4 regions of the 16S rRNA gene were amplified using primers 341F (5′-CCTACGGGNGGCWGCAG-3′) and 805 R (5′-GACTACHVGGGTATCTAATCC-3′).^34^ Each sample was amplified in triplicate in a 25 μL PCR reaction containing 12.5 μL KAPA HiFi HotStart ReadyMix, 1 μL each of 10 μM forward and reverse primers, 2 μL template DNA, and nuclease-free water. PCR conditions were as follows: initial denaturation at 95 °C for 3 min; 25 cycles of 95 °C for 30 s, 55 °C for 30 s, and 72 °C for 30 s; final extension at 72 °C for 5 min. Amplified products were purified using AMPure XP beads (Beckman, Cat#A63880), and quantified with a Qubit dsDNA HS Assay Kit (Thermo Fisher, Q32851). Negative controls with no amplification products were considered valid. Sequencing libraries were constructed and library fragment size was assessed using an Agilent 2100 Bioanalyzer. Qualified libraries were sequenced on the Illumina NovaSeq 6000 platform (PE250 mode), with approximately 96 samples per run and a minimum sequencing depth of 50,000 paired-end reads per sample.

**Public dataset retrieval.** Public datasets were systematically retrieved from the NCBI SRA database (up to March 2024) using the keywords “gestational diabetes”, “fecal microbiome”, and “16S”. Eighteen datasets were initially identified. Inclusion criteria were: (1) availability of raw fecal 16S rRNA sequencing data; (2) clear GDM/non-GDM group designation with clinical metadata; (3) sufficient sample size and sequencing depth (>10,000 reads/sample); (4) absence of severe contamination or batch effects. Eight datasets (e.g., PRJEB58050, PRJNA945212) met all criteria. Metadata fields (age, gestational age, region, pregnancy outcome) were curated and cross-validated independently by two investigators.

### 16S rRNA data processing and taxonomic assignment

Raw sequences were processed using QIIME2 (v2024.10.1).^35^ Steps included merging paired-end read, chimera removal and denoising with DADA2. Only samples with sequencing depth >10,000 were retained. Taxonomic classification was performed using the Silva 138.1 database and the q2-feature-classifier plugin.

#### Linear discriminant analysis effect size (LEfSe)

LEfSe was employed to identify differentially abundant microbial taxa between the GDM and non-GDM groups. First, the non-parametric factorial Kruskal-Wallis sum-rank test was utilized to detect features with significant differential abundance (*P* < 0.05). Subsequently, LDA was performed to estimate the effect size of each differentially abundant feature. Taxa achieving a logarithmic LDA score >2.0 were considered to have statistically significant discriminative power.

### Machine learning modeling and performance evaluation

Maternal fecal and salivary cohorts were randomly divided into training and validation sets at a 7:3 ratio, with stratified sampling to preserve balanced proportions of GDM and non-GDM subjects (**Extended Data Figure 18**). Feature selection was first performed using L1-regularized logistic regression (scikit-learn v1.5.2, C = 0.1, random_state = 42) on OTU abundance data, which had been standardized to zero mean and unit variance. Subsequently, selected OTU features were used to train a random forest classifier, with hyperparameters (number of trees [n_estimators: 100–1000], maximum tree depth [max_depth: 3–20], etc.) optimized by grid search (GridSearchCV) combined with fivefold stratified cross-validation. The best hyperparameter combination, as determined by cross-validation performance, was used in the final model. In addition, ensemble modeling was performed using AutoGluon (v1.2), which automatically integrated multiple base learners including random forest, LightGBM, and XGBoost. Class imbalance was addressed using the Synthetic Minority Over-sampling Technique (SMOTE; imblearn v0.12.3, k_neighbors = 5, random_state = 42) with the ‘auto’ sampling strategy to ensure a balanced (1:1) ratio. All analysis scripts are publicly available on GitHub. Model performance was primarily assessed using the AUC, with additional evaluation using accuracy, precision, recall, and F1-score where appropriate. All models were evaluated on independent holdout test sets.^36^

### Definition of diagnostic grey zone and subgroup assignment

Based on the final L1+RF classifier, validation samples were categorized into three groups according to predicted probability: “Clear_NonGDM” (*P* ≤ 0.3, clinically non-GDM), “Clear_GDM” (*P* ≥ 0.7, clinically GDM), and “grey zone” (0.4 ≤ *P* ≤ 0.6).^37^ Grey zone samples were cross-validated with the AutoGluon model for consistency. All group assignments were double-blinded, with independent interpretation by clinical and bioinformatics teams.

### Microbial network construction and stability analysis

OTU abundance matrices were rarefied and normalized, with OTUs of prevalence <10% or mean abundance <0.00005 filtered out. Centered log-ratio (CLR) transformation was applied. OTU–OTU associations were calculated using Spearman’s rank correlation (scipy v1.11.0), with significant associations defined as FDR-adjusted *P* < 0.05 and |ρ| ≥ 0.3. Co-occurrence networks for GDM and non-GDM groups were constructed and visualized using Gephi (v0.10.1). Network stability was defined as described in the main text. For the core subnetwork “XNP_Guild1”, network metrics—including mean degree, clustering coefficient, shortest path length, and centrality—were calculated, with significance evaluated by comparison to 1,000 random networks (null models) using Z-scores. Robustness was assessed via node removal simulations (by degree centrality and random order), quantified as changes in the largest connected component.^38^

### Functional prediction of the gut microbiota

The functional potential of the gut microbiota was inferred from the 16S rRNA gene sequencing data using PICRUSt2 (Phylogenetic Investigation of Communities by Reconstruction of Unobserved States 2; https://github.com/picrust/picrust2). Using the feature table and representative sequences as input files, the functional compositions were mapped to the Kyoto Encyclopedia of Genes and Genomes (KEGG) database (https://www.genome.jp/kegg/).

### Cross-country meta-analysis

For all country-specific cohorts, samples were dichotomized by the median abundance of “XNP_Guild1”. Log odds ratios (LogORs) and standard errors (SEs) for GDM incidence were calculated. A fixed-effect model was implemented using PyMeta, with heterogeneity assessed by the I² statistic. Combined effect sizes, confidence intervals, and *P* values are presented in the main text and Supplementary Materials.^39^

### Multivariable regression for preterm neonatal outcomes

Multivariable linear regression models were constructed to assess associations between maternal “XNP_Guild1” abundance (high *vs.* low) and continuous clinical outcomes of extremely preterm neonates. Covariates included neonatal postnatal age, gestational age at birth. Analyses were performed using statsmodels (v0.14.4), with two-sided hypothesis testing and *P* < 0.05 considered significant.

## Statistical analysis

All statistical analyses were performed in Python (v3.12.7). Correlation analyses, non-parametric tests (Kruskal-Wallis H test), and multivariable regressions were conducted using scipy (v1.15.3) and statsmodels (v0.14.4). Multiple testing correction was performed using the Benjamini-Hochberg method. Figures were generated with matplotlib (v3.10.3) and seaborn (v0.13.2).

## Supplementary Material

Supplementary_figure_20251230_cleaned.docx Supplementary_figure_20251230_cleaned.docx

Supplementary_table_20251104_cleaned.docx Supplementary_table_20251104_cleaned.docx

## Data Availability

All datasets supporting this study are available from the corresponding author upon reasonable request; in addition, the following NCBI BioProject records are publicly accessible: PRJNA788699 (https://www.ncbi.nlm.nih.gov/bioproject/?term=PRJNA788699), PRJEB58050 (https://www.ncbi.nlm.nih.gov/bioproject/?term=PRJEB58050), PRJNA421371 (https://www.ncbi.nlm.nih.gov/bioproject/?term=PRJNA421371), PRJNA876126 (https://www.ncbi.nlm.nih.gov/bioproject/?term=PRJNA876126), PRJNA945212 (https://www.ncbi.nlm.nih.gov/bioproject/?term=PRJNA945212), PRJNA963229 (https://www.ncbi.nlm.nih.gov/bioproject/?term=PRJNA963229), PRJNA715072 (https://www.ncbi.nlm.nih.gov/bioproject/?term=PRJNA715072), and PRJNA401977 (https://www.ncbi.nlm.nih.gov/bioproject/?term=PRJNA401977). Original code has been deposited at Github and is publicly available through https://github.com/DHLY-SMU/code.
