# Supplementary material for: Dynamic involvement of the core gut microbiome XNP_Guild1 in the evolution of gestational diabetes mellitus
Source: Gut Microbes. 2026 Jan 31;18(1):2623353. doi: 10.1080/19490976.2026.2623353 (PMC12867416; doi:10.1080/19490976.2026.2623353)
Supplement: Supplementary_table_20251104_cleaned.docx [file KGMI_A_2623353_SM2628.docx]

**Dynamic involvement of the core gut microbiome XNP_Guild1 in the evolution of gestational diabetes mellitus**

Hualongyue Du^1,#^, Qiaoxi Lin^2,#^, Xiaojing He^1,#^, Bin Yang^3^, Yiyao Huang^1^, Qianbei Li^1^, Yudi Wang^1^, Ruijing Wen^1^, Wenlong Lin^1^, Shenghui Li^4^, Lei Zheng^1,^*, Zihao Ou^1,2,^*

^1^ Department of Laboratory Medicine, Nanfang Hospital, Southern Medical University, Guangzhou, 510515, China.

^2^ Guangdong Provincial Clinical Research Center for Laboratory Medicine, Guangzhou, 510260, China.

^3^ Center for Infectious Diseases, Vision Medicals Co., Ltd, Guangzhou, 510515, China.

^4^ Puensum Genetech Institute, Wuhan, 430076, China.

^#^ These authors contributed equally

^*^ Correspondence: [ozh_xnp@smu.edu.cn](mailto:ozh_xnp@smu.edu.cn) (Zihao Ou); nfyyzhenglei@smu.edu.cn (Lei Zheng)

**Table 1. Maternal gestational status and fecal microbiota composition from Dataset PRJNA788699**

|  | GDM feces (*n*=20) | non-GDM feces (*n*=55) |
| --- | --- | --- |
| ^a^T1 |  |  |
| Obese | 4 | 2 |
| Preobese | 4 | 10 |
| Normal | 3 | 12 |
| Mildunderweight | 0 | 2 |
| ^b^T3 |  |  |
| Obese | 3 | 4 |
| Preobese | 4 | 9 |
| Normal | 2 | 14 |
| Mildunderweight | 0 | 2 |

Maternal BMI categories and their distribution among GDM and non-GDM groups at early (aT1, <13 weeks) and late (bT3, >27 weeks) gestational stages. Data are presented as counts. aT1, gestational age <13 weeks; bT3, gestational age >27 weeks.

**Table 2. Clinical characteristics of maternal and infant samples from Dataset PRJEB58050**

|  | GDM (*n*=326) | non-GDM (*n*=1153) | *P* value |
| --- | --- | --- | --- |
| Feces |  |  |  |
| BMI (kg/m^2^) | 27.55 ± 7.57 (n= 152) | 22.86 ± 4.07  (n=575) | <0.001 |
| Age (year) | 32.71 ± 4.18  (n=154) | 32.23 ± 4.11  (n=592) | 0.203 |
| Total specimens | 154 | 592 |  |
| Saliva |  |  |  |
| BMI (kg/m^2^) | 27.49 ± 6.56  (n=151) | 22.59 ± 3.77  (n=504) | <0.001 |
| Age (year) | 33.30 ± 4.02  (n=151) | 31.87 ± 4.28  (n=538) | <0.001 |
| Total specimens | 151 | 538 |  |
| Infant feces |  |  |  |
| BMI (kg/m^2^) | Not collected | Not collected |  |
| Age (year) | Not collected | Not collected |  |
| Total specimens | 21 | 23 |  |

Summary of maternal and infant clinical characteristics, including BMI and age, and total number of fecal and salivary specimens, stratified by GDM and non-GDM groups. Data are presented as mean ± s.d. unless otherwise indicated. *P* values were calculated using Welch's t-test .

**Table 3. Maternal fecal and clinical characteristics from Dataset PRJNA421371**

|  | GDM feces (*n=*21) | non-GDM feces (*n*=39) | *P* value |
| --- | --- | --- | --- |
| BMI (kg/m^2^) | 30.84 ± 5.41 | 25.41 ± 4.35 | <0.001 |
| Age (year) | 35.86 ± 3.66 | 28.03 ± 5.01 | <0.001 |
| Race | | | 0.802 |
| Mixed-Race | 9 | 20 |  |
| White | 9 | 15 |  |
| Black | 3 | 4 |  |

Maternal clinical characteristics and fecal sample counts for GDM and non-GDM groups. Data are presented as mean ± s.d. or n (%). *P* values were calculated using Welch's t-test.

**Table 4. Clinical characteristics of infants and mothers from Dataset PRJNA976126**

| Variable | Value for | | *P* Value |
| --- | --- | --- | --- |
|  | GDM group (*n*=60) non-GDM group (*n*=60) | |  |
| Maternal |  | | |
| Age (year) | 28.9 ± 3.66 | 28.5 ± 3.96 | 0.551 |
| Prepregnancy BMI (kg/m^2^) | 21.9 ± 3.48 | 20.9 ± 2.62 | 0.099 |
| OGTT_FBG | 4.50 ± 0.70 | 4.06 ± 0.37 | <0.001 |
| OGTT_1h | 8.05 ± 2.09 | 6.54 ± 1.52 | <0.001 |
| OGTT_2h | 6.93 ± 1.58 | 5.87 ± 1.08 | <0.001 |
| Gestational age (week) | 39.2 ± 1.27 | 39.2 ± 1.01 | 0.887 |
| Parity [no. (%)] |  |  | 1.000 |
| Nulliparae | 47 (78.3) | 46 (76.7) |  |
| Multiparae | 13 (21.7) | 14 (23.3) |  |
| Passive smoking [no. (%)] |  |  | 0.561 |
| Never | 42 (70.0) | 38 (63.3) |  |
| Ever | 18 (30.0) | 22 (36.7) |  |
| Alcohol drinking [no. (%)] |  |  | 0.439 |
| Never | 58 (96.7) | 55 (91.7) |  |
| Ever | 2 (3.33) | 5 (8.33) |  |
| Neonatal |  |  | 1.000 |
| Delivery mode [no. (%)] |  |  |  |
| Cesarean | 44 (73.3) | 44 (73.3) |  |
| Vaginal | 16 (26.7) | 16 (26.7) |  |
| Sex [no.(%)] |  |  |  |
| Boy | 35 (58.3) | 36 (60.0) |  |
| Girl | 25 (41.7) | 24 (40.0) |  |
| Birth weight (g) | 3,476 (400) | 3,361 (360) | 0.111 |
| Breastfeeding [no. (%)] |  |  | 0.777 |
| Exclusive breastfeeding | 8 (13.3) | 6 (10.0) |  |
| Mixed feeding | 52 (86.7) | 53 (88.3) |  |
| Formula feeding | 0 (0.00) | 1 (1.67) |  |
| Weight at 12 mo old (kg) | 10.4 (1.04) | 10.1 (0.93) | 0.048 |
| Height at 12 mo old (cm) | 76.6 (2.45) | 76.8 (2.93) | 0.782 |
| BMI at 12 mo old (kg/m^2^) | 17.8 (1.24) | 17.1 (1.27) | 0.006 |
| BMI Z-score at 12 mo old | 0.77 (0.77) | 0.31 (0.95) | 0.004 |

Comparison of maternal and neonatal characteristics in the GDM and non-GDM groups, including OGTT results, delivery mode, birth outcomes, and feeding status. Data are presented as mean ± s.d., n (%), or median (IQR) as appropriate. *P* values were calculated using Student’s t-test or Fisher’s exact test.

**Table 5. Maternal clinical information from Dataset PRJNA945212**

|  |  | GDM feces (*n*=87) | non-GDM feces (*n*=16) | *P* value |
| --- | --- | --- | --- | --- |
| ^a^T1 |  |  |  | 0.758 |
| Chinese | | 22 | 6 |  |
| Indian | | 11 | 5 |  |
| Malaysian | | 13 | 5 |  |
| ^b^T2 |  |  |  | NA |
| Chinese | | 22 | 0 |  |
| Indian | | 9 | 0 |  |
| Malaysian | | 10 | 0 |  |

Summary of maternal clinical data collected at different gestational stages for GDM and non-GDM groups.
aT1, 24–28 weeks of gestation; bT2, 36–40 weeks of gestation. P value was calculated for the overall ethnic distribution at T1 using Fisher's exact test.

**Table 6. Infant clinical information from Dataset PRJNA945212**

|  |  | Infant GDM feces (*n*=63) | | Infant non-GDM feces (*n*=25) | |
| --- | --- | --- | --- | --- | --- |
|  |  | male | female | male | female |
| First week |  |  |  |  |  |
| Chinese | | 9 | 8 | 4 | 3 |
| Indian | | 4 | 4 | 2 | 0 |
| Malaysian | | 5 | 5 | 3 | 2 |
| Sixth week |  |  |  |  |  |
| Chinese | | 9 | 6 | 2 | 2 |
| Indian | | 5 | 3 | 3 | 1 |
| Malaysian | | 3 | 4 | 1 | 2 |

Infant sex and ethnicity distribution at the first and sixth week postpartum in GDM and non-GDM groups. Data are presented as counts.

**Table 7. Maternal fecal sample-related clinical characteristics from Dataset PRJNA963229**

|  | GDM feces (*n*=72) | non-GDM feces (*n*=61) |
| --- | --- | --- |
| ^a^T1 | 27 | 25 |
| ^b^T2 | 27 | 28 |
| ^c^T3 | 18 | 8 |

Maternal clinical characteristics and fecal sample distribution at different time points. Data are presented as counts.
aT1, 11.85 ± 1.05 weeks of gestation; bT2, 30.50 ± 2.59 weeks; cT3, 6–8 weeks postpartum.

**Table 8. Clinical characteristics of the maternal gestational fecal early warning cohort (Nanfang Hospital)**

| Variables | GDMfeces (*n*=15) | non-GDMfeces (*n*=42) | *P* value |
| --- | --- | --- | --- |
| BMI (kg/m^2^) | 24.69 ± 2.97 | 21.96 ± 2.82 | 0.005 |
| Age (year) | 31.13 ± 4.84 | 27.83 ± 3.41 | 0.025 |
| Fasting glucose (mmol/L) | 8.61 ± 0.87 | 5.06 ± 0.42 | < 0.001 |
| Delivery week (week) | 39.20 ± 1.52 | 39.07 ± 0.75 | 0.757 |

Maternal clinical characteristics, including BMI, age, fasting glucose, and delivery week, in GDM and non-GDM groups. Data are presented as mean ± s.d. *P* values were calculated using Welch's t-test

**Table 9. L1+RF model prediction intervals in the validation set**

| GDM Predicted Probability Interval | Total Sample Size (*n* = 258) | Actual GDM  Count (*n* = 99) | Actual non-GDM Count (*n* = 159) | Model Prediction Error Rate |
| --- | --- | --- | --- | --- |
| [0.0 , 0.1] | 0 | 0 | 0 |  |
| [0.1 , 0.2] | 0 | 0 | 0 |  |
| [0.2 , 0.3] | 62 | 9 | 53 | 14.52% |
| [0.3 , 0.4] | 90 | 16 | 74 | 17.78% |
| [0.4 , 0.5] | 49 | 25 | 24 | 51.02% |
| [0.5 , 0.6] | 6 | 1 | 5 | 83.3% |
| [0.6 , 0.7] | 4 | 2 | 2 | 50% |
| [0.7 , 0.8] | 7 | 6 | 1 | 14.29% |
| [0.8 , 0.9] | 20 | 20 | 0 | 0% |
| [0.9 , 1.0] | 20 | 20 | 0 | 0% |

Distribution of samples, true GDM/non-GDM counts, and model error rates across different predicted probability intervals for the L1+RF model in the validation set (n = 258).
Error rate: proportion of misclassified samples in each probability interval.

**Table 10. Comparison of Topological Properties of GDM feces and non-GDM feces Networks**

| Network Type | Nodes | Edges | Density | Proportion of Negative Edges |
| --- | --- | --- | --- | --- |
| non-GDM Netweork | 197 | 6054 | 0.3136 | 0.159 |
| GDM Network | 211 | 5199 | 0.2347 | 0.04 |

The GDM and non-GDM networks were constructed based on the fecal training set, where edges represent significant Spearman correlations between OTUs (absolute correlation coefficient |ρ| ≥ 0.3 and FDR < 0.05).

*Node: A microbial unit.*

*Edge: The relationship connecting two nodes.*

*Network Density: The degree of connectedness in a network.*

**Table 11. Internal centrality of XNP_Guild1**

| OTU | Degree Centrality | Edges Betweenness Centrality |
| --- | --- | --- |
| OTU-46 | 1.000 | 0.352 |
| OTU-88 | 0.938 | 0.227 |
| OTU-181 | 0.625 | 0.044 |
| OTU-453 | 0.500 | 0.018 |
| OTU-289 | 0.438 | 0.013 |

The internal centrality of XNP_Guild 1 is based on the subnetwork within the stable network that is composed solely of the XNP_Guild 1 nodes and the edges that exist between them.

**Table 12. Enrichment of XNP_Guild1 in different countries**

| Country | Median GDM | Mean GDM | Median non-GDM | Mean non-GDM | *P* value |
| --- | --- | --- | --- | --- | --- |
| Brazil | 0.009814 | 0.010286 | 0.005587 | 0.007094 | 0.24 |
| Israel | 0.001992 | 0.002599 | 0.001069 | 0.001858 | 0.01 |
| Malaysia | 0.073268 | 0.080983 | 0.031192 | 0.035056 | 0.01 |

Mann-Whitney U test analysis was performed on the fecal validation set by country, after handling outliers using the IQR method.

**Table 13. Metagenomics sample numbers in Dataset PRJNA401977**

|  | GDMfeces | non-GDMfeces |
| --- | --- | --- |
| Total specimens | 30 | 25 |

Summary of the number of maternal fecal samples analyzed by metagenomics sequencing in GDM and non-GDM groups.

**Table 14. Comparison of Topological Properties of Infant GDM feces and Infant non-GDM feces Networks**

| Network Type | Nodes | Edges | Density | Proportion of Negative Edges |
| --- | --- | --- | --- | --- |
| non-GDM Netweork | 28 | 189 | 0.5000 | 0.085 |
| GDM Network | 27 | 106 | 0.3020 | 0.085 |

The GDM and non-GDM networks were constructed based on the Infant fecal training set, where edges represent significant Spearman correlations between OTUs (absolute correlation coefficient |ρ| ≥ 0.3 and FDR < 0.05).

**Table 15. Infant GDM feces network**

| OTU | DegreeCentrality | EdgesBetweennessCentrality |
| --- | --- | --- |
| OTU-715 | 0.615 | 0.280 |
| OTU-101 | 0.423 | 0.134 |
| OTU-44 | 0.346 | 0.061 |
| OTU-425 | 0.308 | 0.059 |
| OTU-5 | 0.385 | 0.054 |
| OTU-55 | 0.308 | 0.046 |
| OTU-76 | 0.231 | 0.036 |
| OTU-227 | 0.385 | 0.034 |
| OTU-46 | 0.385 | 0.034 |
| OTU-130 | 0.269 | 0.031 |

The GDM network was constructed based on the Infant fecal training set, where edges represent significant Spearman correlations between OTUs (absolute correlation coefficient |ρ| ≥ 0.3 and FDR < 0.05).

*Degree Centrality: The number of direct connections a node has.*

*Betweenness Centrality: The frequency with which a node appears on all shortest paths in the network.*

**Table 16. Infant birth and early clinical outcomes from Dataset PRJNA715072**

|  | Female (*n*=291) | Male (*n*=230) | *P* value |
| --- | --- | --- | --- |
| Birth_head_circumference (cm) | 22.80 ± 1.56 | 23.34 ± 1.65 | < 0.001 |
| Birth_length (cm) | 32.26 ± 2.51 | 33.20 ± 2.13 | < 0.001 |
| Birth_weight (g) | 726.81 ± 160.51 | 773.21 ± 127.69 | < 0.001 |
| Days_in_hospital (day) | 101.22 ± 49.51 | 92.14 ± 17.43 | 0.004 |
| GA (week) | 25.52 ± 1.40 | 25.51 ± 1.32 | 0.933 |
| Apg10 | 8.88 ± 0.38 | 8.75 ± 0.99 | 0.060 |
| Apg5 | 8.56 ± 0.54 | 8.26 ± 1.30 | 0.001 |
| has_EOS_neoKISS |  |  | < 0.001 |
| Yes | 131 | 156 |  |
| No | 159 | 73 |  |
| has_LOS_neoKISS |  |  | 0.902 |
| Yes | 217 | 169 |  |
| No | 73 | 60 |  |
| Patent Ductus Arteriosus |  |  | < 0.001 |
| Yes | 141 | 83 |  |
| No | 141 | 146 |  |

Comparison of birth and early postnatal parameters between male and female infants, including head circumference, length, weight, gestational age, hospital stay, Apgar scores, and incidence of early/late-onset sepsis (neoKISS definitions). Data are presented as mean ± s.d. or n (%). *P* values were calculated using Student’s t-test or Fisher’s exact test.

**Table 17. Abundance of XNP_Guild1 in the Nanfang Hospital Early Warning Cohort**

|  | GDM feces  (n = 15) | non-GDM feces (n = 42) | *P* value |
| --- | --- | --- | --- |
| Median | 0.0026 | 0.0018 | 0.5818 |
| Mean | 0.0090 | 0.0142 |  |

Mann-Whitney U test analysis was performed on the Nanfang Hospital Early Warning Cohort.

**Table 18. Taxonomic composition of the core functional guild XNP_Guild1.**

| OTU ID | Taxonomic Assignment |
| --- | --- |
| OTU-46 | *g__Escherichia* |
| OTU-88 | *g__Olsenella* |
| OTU-181 | *g__Sellimonas* |
| OTU-453 | *g__[Ruminococcus]_gnavus_group* |
| OTU-289 | *g__Weissella* |
| OTU-94 | *g__Occallatibacter* |
| OTU-33 | *o__Oscillospirales* |
| OTU-64 | *g__Anaerotruncus* |
| OTU-850 | *g__Eubacterium* |
| OTU-115 | *g__Eisenbergiella* |
| OTU-260 | *g__Negativibacillus* |
| OTU-413 | *g__Enterococcus* |
| OTU-68 | *g__Acidaminococcus* |
| OTU-257 | *g__Tyzzerella* |
| OTU-53 | *g__Megamonas* |
| OTU-24 | *g__Staphylococcus* |
| OTU-99 | *g__Intestinimonas* |

Taxonomy assigned via QIIME 2 against the SILVA 138.1 database.
