# Supplementary material for: Dynamic involvement of the core gut microbiome XNP_Guild1 in the evolution of gestational diabetes mellitus
Source: Gut Microbes. 2026 Jan 31;18(1):2623353. doi: 10.1080/19490976.2026.2623353 (PMC12867416; doi:10.1080/19490976.2026.2623353)
Supplement: Supplementary_figure_20251230_cleaned.docx [file KGMI_A_2623353_SM2629.docx]

**Dynamic involvement of the core gut microbiome XNP_Guild1 in the evolution of gestational diabetes mellitus**

Hualongyue Du^1,#^, Qiaoxi Lin^2,#^, Xiaojing He^1,#^, Bin Yang^3^, Yiyao Huang^1^, Qianbei Li^1^, Yudi Wang^1^, Ruijing Wen^1^, Wenlong Lin^1^, Shenghui Li^4^, Lei Zheng^1,^*, Zihao Ou^1,2,^*

^1^ Department of Laboratory Medicine, Nanfang Hospital, Southern Medical University, Guangzhou, 510515, China.

^2^ Guangdong Provincial Clinical Research Center for Laboratory Medicine, Guangzhou, 510260, China.

^3^ Center for Infectious Diseases, Vision Medicals Co., Ltd, Guangzhou, 510515, China.

^4^ Puensum Genetech Institute, Wuhan, 430076, China.

^#^ These authors contributed equally

^*^ Correspondence: [ozh_xnp@smu.edu.cn](mailto:ozh_xnp@smu.edu.cn) (Zihao Ou); nfyyzhenglei@smu.edu.cn (Lei Zheng)

**Extend Figure 1.** Phylogenetic tree illustrating the taxonomic composition and relative abundance of microbial taxa in fecal samples from all study participants.

**Extend Figure 2.** Phylogenetic tree showing the taxonomic structure and relative abundance of salivary microbiota in all samples analyzed.

**Extend Figure 3.** Co-occurrence network depicting significant correlations among OTUs in the fecal microbiota of the GDM group.

**Extend Figure 4.** Network representation of significant OTU co-occurrences in fecal samples from the non-GDM group.

.

**Extend Figure 5.** Stable subnetworks extracted from the GDM and non-GDM co-occurrence networks.

**Extend Figure 6.** Relative abundance and structural features of XNP_Guild1 in (A) the stable subnetwork and (B) the GDM-specific co-occurrence network.

**Extend Figure 7.** Alterations in the co-occurrence patterns and centrality of *Escherichia* (OTU-46) during the transition from non-GDM (A) to GDM (B).

**Extend Figure 8.** A, Relative abundance of XNP_Guild1 in an independent validation cohort (n = 99 (GDM), n = 159 (non-GDM)).

B, Abundance of XNP_Guild1 in samples from Brazil, Israel, and Malaysia.

C, Changes in XNP_Guild1 abundance across pregnancy trimesters (n = 27 (GDM T1), n = 27 (GDM T2), n = 18 (GDM T3)).

D, Differential abundance of key metabolic pathways in *Akkermansia* between GDM and non-GDM.

E, Differential abundance of functional pathways in *Lachnoclostridium* between GDM and controls.

**Extend Figure 9.** Microbial co-occurrence network based on meconium samples from infants born to non-GDM mothers.

**Extend Figure 10.** Microbial co-occurrence network based on meconium samples from infants born to GDM mothers.

**Extend Figure 11.** Stable subnetworks extracted from meconium microbiota of GDM and non-GDM neonates.

**Extend Figure 12.** Comparison of neonatal Apgar scores between high and low XNP_Guild1 abundance groups. A, Apgar scores at 5 minutes (Apg5); B, Apgar scores at 10 minutes (Apg10).

**Extend Figure 13.** Total abundance of XNP_Guild1 in fecal samples from Clear_NonGDM (n = 53), grey zone (n = 55), and Clear_GDM (n = 46) participants. Data are presented as mean ± s.d.; statistical significance determined by two-tailed unpaired t-test.

**Extend Figure 14.** Interaction network showing the relationships between grey zone indicator OTUs and XNP_Guild1 members in fecal samples from the Clear_NonGDM group.

**Extend Figure 15.** Network analysis illustrating interactions among grey zone indicator OTUs and XNP_Guild1 in grey zone fecal samples.

**Extend Figure 16.** Interaction network showing connections between grey zone indicator OTUs and XNP_Guild1 in Clear_GDM fecal samples.

**Extend Figure 17.** Network comparison showing dynamic alterations in the connectivity between XNP_Guild1 and key grey zone variables within the Clear_GDM group.

**Extended Figure 18.** The flowchart illustrates the partitioning of included datasets into training and validation sets.
